# Regulation of myeloid and lymphoid cell development by O-glycans on Notch

**DOI:** 10.3389/fmolb.2022.979724

**Published:** 2022-11-04

**Authors:** Pamela Stanley, Ankit Tanwar

**Affiliations:** Department of Cell Biology, Albert Einstein College Medicine, New York, NY, United States

**Keywords:** Notch signaling, O-fucose, O-GlcNAc, Fringe, HSCs, lymphoid, myeloid

## Abstract

Notch signaling *via* NOTCH1 stimulated by Delta-like ligand 4 (DLL4) is required for the development of T cells in thymus, and NOTCH2 stimulated by Notch ligand DLL1 is required for the development of marginal zone (MZ) B cells in spleen. Notch signaling also regulates myeloid cell production in bone marrow and is an essential contributor to the generation of early hematopoietic stem cells (HSC). The differentiation program in each of these cellular contexts is optimized by the regulation of Notch signaling strength by O-glycans attached to epidermal growth factor-like (EGF) repeats in the extracellular domain of Notch receptors. There are three major types of O-glycan on NOTCH1 and NOTCH2 - O-fucose, O-glucose and O-GlcNAc. The initiating sugar of each O-glycan is added in the endoplasmic reticulum (ER) by glycosyltransferases POFUT1 (fucose), POGLUT1/2/3 (glucose) or EOGT (GlcNAc), respectively. Additional sugars are added in the Golgi compartment during passage through the secretory pathway to the plasma membrane. Of particular significance for Notch signaling is the addition of GlcNAc to O-fucose on an EGF repeat by the Fringe GlcNAc-transferases LFNG, MFNG or RFNG. Canonical Notch ligands (DLL1, DLL4, JAG1, JAG2) expressed in stromal cells bind to the extracellular domain of Notch receptors expressed in hematopoietic stem cells and myeloid and lymphoid progenitors to activate Notch signaling. Ligand-receptor binding is differentially regulated by the O-glycans on Notch. This review will summarize our understanding of the regulation of Notch signaling in myeloid and lymphoid cell development by specific O-glycans in mice with dysregulated expression of a particular glycosyltransferase and discuss how this may impact immune system development and malignancy in general, and in individuals with a congenital defect in the synthesis of the O-glycans attached to EGF repeats.

## Introduction

**FIGURE 1:**
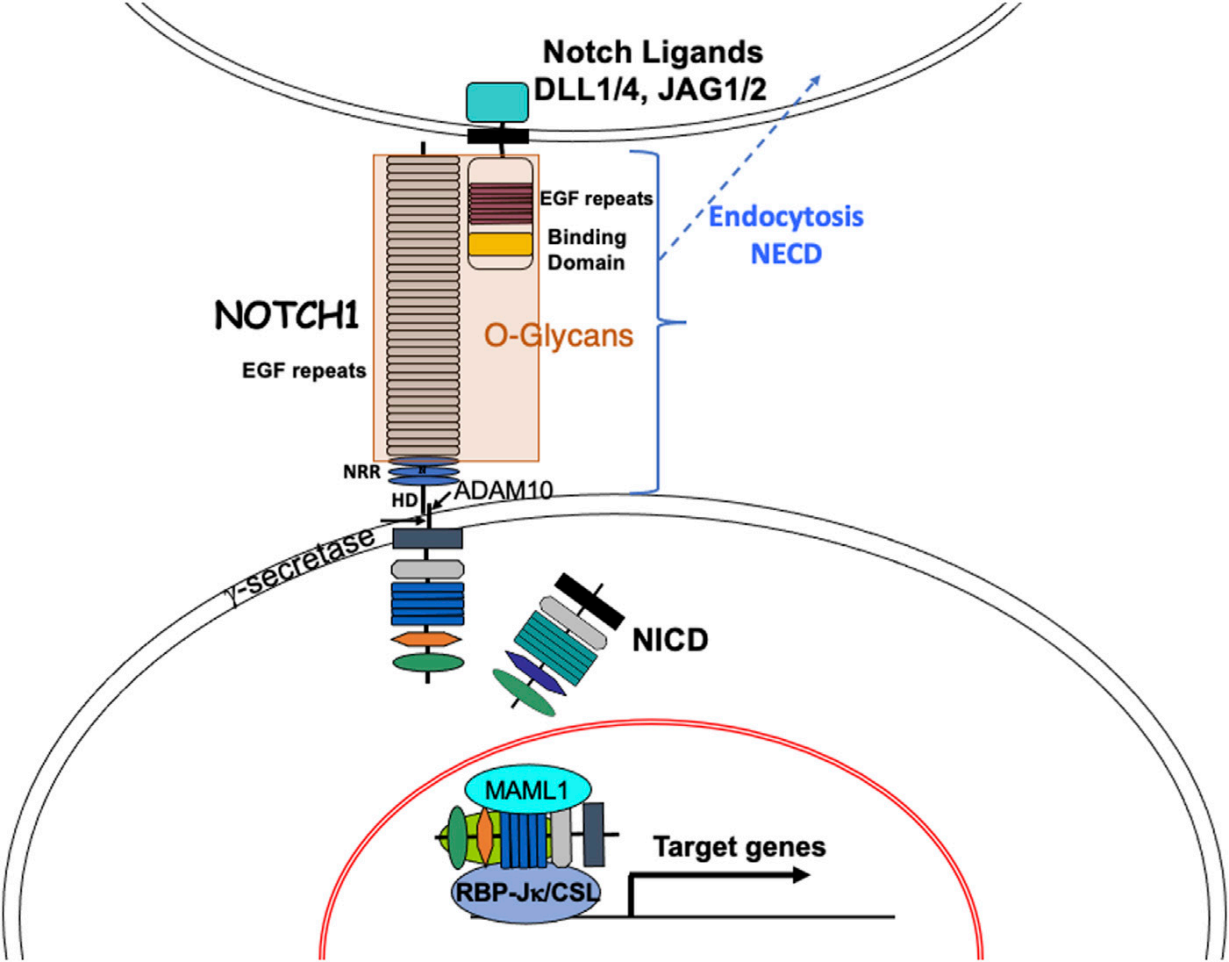
The Notch signaling pathway. The basic components of the Notch signaling pathway are shown. Canonical Notch ligands DLL1, DLL4, JAG1 and JAG2 in a signal-sending cell contact heterodimeric Notch receptors (e. g. NOTCH1) in a signal-receiving cell to initiate Notch signaling. Binding of ligand to NOTCH1 is regulated by O-glycans on EGF repeats of the Notch ECD and induces a conformational change that exposes a juxta-membrane cleavage site for the metalloprotease ADAM10. The released Notch ECD is endocytosed into the signal-sending cell. The transmembrane remnant of NOTCH1 is then cleaved within the membrane by gamma-secretase. The released Notch ICD transits to the nucleus where it combines with RBP-Jκ/CSL and transcriptional activators including MAML1 to form a complex that recognizes RBP-Jκ/CSL DNA binding sites in many target genes, thereby activating transcription. Notch signaling target genes include transcriptional suppressors like *Hes* and *Hey* family genes and transcriptional activators like c-Myc. NRR, Notch regulatory domain; HD, heterodimerization domain.

Notch signaling is a highly conserved pathway that plays a pivotal role in the maintenance of tissue development and homeostasis. Defective Notch signaling leads to numerous pathological conditions (Varshney and Stanley, 2018; Zhou et al., 2022). In mammals, there are four Notch receptors (NOTCH1-NOTCH4) and five ligands of the Jagged and Delta-like families (DLL1, DLL3, DLL4 - orthologs to fly Delta; JAG1, JAG2 - orthologs to fly Serrate). Both Notch receptors and the canonical ligands, are transmembrane proteins with an extracellular domain (ECD) that consists primarily of epidermal growth factor-like (EGF) repeats (Figure 1). Many of these EGF repeats harbor consensus sites for the addition of different types of O-glycan to Ser or Thr, which takes place in the endoplasmic reticulum (ER) (Saiki et al., 2021). Classical Notch signaling is induced when Notch receptors interact with Notch ligands in neighboring cells (Kopan, 2012; Hori et al., 2013) (Figure 1). The binding of ligand promotes two proteolytic cleavages of a Notch receptor. The first is catalyzed by an ADAM-family metalloprotease and releases the extracellular domain, which is endocytosed into the ligand-expressing cell (Seib and Klein, 2021). The second cleavage is mediated by γ-secretase, an enzyme complex that contains presenilin, nicastrin, PEN2 and APH1 whose action releases the Notch intracellular domain (ICD), which translocates to the nucleus and cooperates with the DNA-binding protein RBP-Jκ/CSL and a co-activator Mastermind-like 1 (MAML1), to promote transcription and activate Notch target genes (Kovall et al., 2017). There are numerous factors involved in the regulation of Notch signaling, including the expression of Notch ligands in signal-receiving cells that may cause cis-inhibition of Notch receptors, factors affecting secretory pathway trafficking, and the complement of O-glycans attached to the Notch ECD.

**FIGURE 2:**
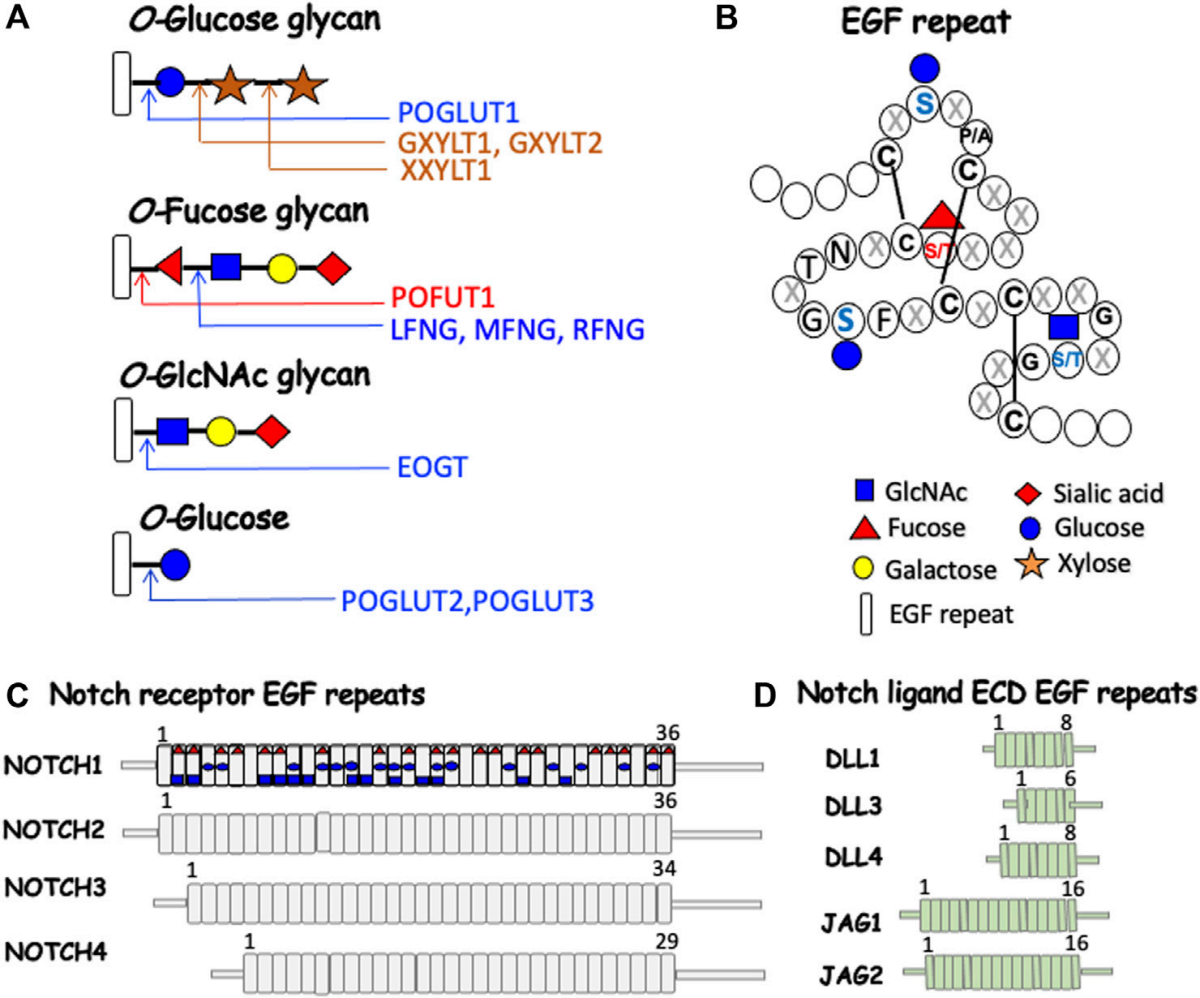
Notch receptor and Notch ligand glycosylation in mammals. **(A)** Each O-glycan predicted to be attached to an EGF repeat with an appropriate consensus sequence is shown. **(B)** A generic EGF repeat of ∼40 amino acids (Campbell et al., 1990) with the relevant sugar attached at the consensus site (Takeuchi et al., 2018a). Amino acids are shown by single letter code or X for any amino acid. The symbol for each sugar and an EGF repeat is shown in the key. The glycosyltransferases responsible for each sugar transfer are given. **(C)** NOTCH1-NOTCH4 ECDs are depicted with their respective number of EGF repeats, many of which can be modified by one or more O-glycans. The predicted O-glycan additions to mouse NOTCH1 are indicated by the respective initiating sugar. **(D)** The ECD of canonical Notch ligands with their respective number of EGF repeats, several of which can be modified by O-glycans.

The major O-glycans found on EGF repeats of Notch receptors and the canonical Notch ligands are initiated by three different sugars: 1) O-fucose is added to Ser/Thr in the consensus site C_2_XXXX(S/T)C_3_ between the second and third Cys residues, where X is any amino acid, and either Ser or Thr accepts a fucose transferred from GDP-fucose by protein O-fucosyltransferase 1 (POFUT1); 2) O-glucose is added to Ser in the consensus site C_1_XSX(P/A)C_2_ where Ser accepts glucose from UDP-Glc transferred by protein O-glucosyltransferase 1 (POGLUT1); and 3) O-GlcNAc added to Ser/Thr in the consensus site C_5_XXGX(S/T)GXXC_6_ where Ser or Thr accepts GlcNAc transferred from UDP-GlcNAc by EGF domain-specific O-linked N-acetylglucosaminyltransferase (EOGT) (Saiki et al., 2021) (Figures 2A,B). The consensus sequence for POGLUT2 and POGLUT3 is distinct from that of POGLUT1 (Takeuchi et al., 2018a) (Figure 2B). The four mammalian Notch receptors contain large numbers of EGF repeats in their respective ECD (Figure 2C), the Notch ligands contain fewer (Figure 2D). The predicted O-glycan occupation of NOTCH1 based on EGF consensus sequences is shown in Figure 2C. Almost all structural studies to determine O-glycan occupancy have been performed on NOTCH1 constructs expressed in cells overexpressing different O-glycan glycosyltransferases. To date there is no O-glycan analysis of a Notch receptor purified directly from a mammalian physiological source. However, species comparisons of O-glycan consensus sites in EGF repeats reveal that some are highly conserved like the O-fucose site in EGF12, and others may vary between species (Haines and Irvine, 2003; Saiki et al., 2021). Each of the initiating sugars can be extended by the addition of other sugars to form a glycan. O-fucose is extended by the transfer of GlcNAc from UDP-GlcNAc by one of three Fringe GlcNAc-transferases termed LFNG, MFNG and RFNG. The GlcNAc may be extended by Gal, which may in turn be extended by sialic acid (Sia) to generate the O-linked tetra-saccharide Sia-Gal-GlcNAc-Fuc-O-EGF. Structural analyses of NOTCH1 overexpressed in HEK293T cells showed that O-fucose consensus sites almost always have a fucose, but the degree of extension of each fucose is variable (Kakuda and Haltiwanger, 2017). In T cells from mouse spleen activated in culture, Fringe modification of NOTCH1 occurs at few O-fucose residues (Matsumoto et al., 2022). O-glucose may be extended by xylose transferred from UDP-Xyl by xylosyltransferases GXYLT1 or GXYLT2, and a second Xyl may be added by XXYLT1 (Yu and Takeuchi, 2019). O-GlcNAc may be extended by Gal and Sia as well as Fuc (Tsukamoto et al., 2022). Several congenital human diseases arise from mutations in genes encoding the glycosyltransferases that modify EGF repeats in Notch receptors and ligands (Varshney and Stanley, 2018; Matsumoto et al., 2021).

In this review, we summarize our current understanding of roles for O-glycans in Notch signaling during hematopoiesis, myeloid and lymphoid cell development in the mouse. We also discuss how different O-glycans may impact immune cell development and malignancy, in general, and in individuals with a congenital defect in the synthesis of the O-glycans attached to Notch receptors.

### Notch signaling in hematopoiesis

The yolk sac is the first organ with hematopoietic potential in the mouse embryo, producing mainly nucleated red blood cells (RBCs) and a few macrophages and megakaryocytes during primitive hematopoiesis, beginning at embryonic day E7.5 (Pajcini et al., 2011; Bigas et al., 2012). Hematopoiesis continues in the embryo at sites of hemogenic endothelium, initially in the aorta-gonad-mesonephros (AGM), which forms HSCs that migrate to the fetal liver, and from there to the bone marrow (BM), to finally give rise to all erythroid, myeloid and lymphoid cells in the adult. Variations in the strength of Notch signaling are crucial for the switch between the formation of endothelial cells (low Notch signal induced by JAG1), and the formation of HSCs (high Notch signal induced by DLL4) from hemogenic endothelium (Gama-Norton et al., 2015; Porcheri et al., 2020). Abolishing Notch signaling has no effect on hematopoiesis in the yolk sac (Pajcini et al., 2011; Bigas et al., 2012). However, signaling *via* NOTCH1, but not NOTCH2, is required for the generation of HSCs in the embryo, although not for their proliferation or maintenance in the adult (Kumano et al., 2003; Souilhol et al., 2016). On the other hand, various experimental approaches that increase Notch signaling in HSCs lead to increased proliferation and expansion of the stem cell pool (Lampreia et al., 2017). Most recently, conditional deletion of RBP-Jκ in HSC has shown that Notch signaling, while not required for the generation or maintenance of long- or short-term HSCs, is important for HSC self-renewal after irradiation or chemotherapeutic stress (Lakhan and Rathinam, 2021).

Roles for individual Notch receptors and ligands in hematopoiesis have been identified by conditional knockout strategies in the mouse. Overall requirements of Notch signaling have been investigated by inactivation of downstream transcription factors including RBP-Jκ, that are required for signaling through all canonical Notch receptor/ligand interactions (Kovall et al., 2017). Inactivation of *Notch1* in HSCs using *Mx1*-Cre revealed that NOTCH1 is essential for the development of T cells in the thymus (Radtke et al., 1999; MacDonald et al., 2001). Conditional deletion of *Notch1* in thymic T cells using a CD4-Cre revealed requirements for NOTCH1 in the generation of T cells involved in innate immunity in liver, intestine and spleen (Chennupati et al., 2016). A role for NOTCH2 in early T cell development was recently identified in co-culture investigations of *Notch1* and *Notch2* following inactivation in bone marrow progenitors (Romero-Wolf et al., 2020). In elegant rescue experiments of HSCs conditionally-deleted for RBP-Jκ, the downstream effector of Notch signaling, a block in the development of thymus-seeking T cell progenitors in bone marrow was identified (Chen et al., 2019).

Experiments to determine roles for Notch ligands in thymic stromal cells used *Foxn1*-Cre to identify DLL4 as essential for stimulating Notch signaling in the thymus (Hozumi et al., 2008; Koch et al., 2008). However, recent rescue experiments have shown that transgenic *Dll1* can substitute for *Dll4* when *Dll4* is deleted, and can stimulate NOTCH1 and NOTCH2 to promote T cell development in thymus (Hirano et al., 2022). Immunochemistry shows that DLL1, DLL4, JAG1 and JAG2 Notch ligands are expressed in thymus in a regio-specific manner which may be important in controlling Notch signaling strength in different regions of the thymus (Felli et al., 1999; Garcia-Leon et al., 2018).

**FIGURE 3:**
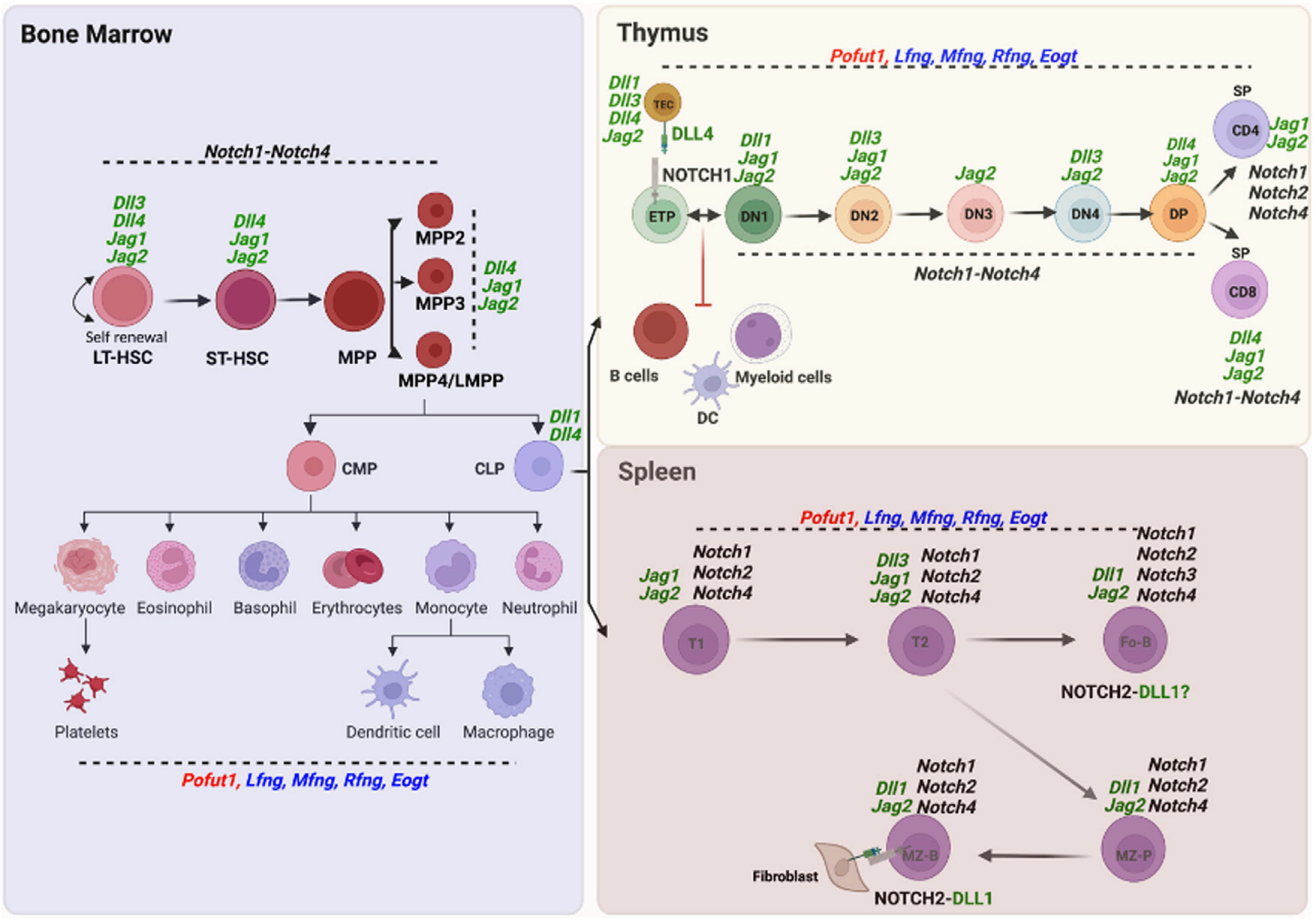
Expression of O-glycan glycosyltransferase, Notch receptor and Notch ligand genes in hematopoietic cells. Expression of the O-glycan glycosyltransferase, Notch receptor and canonical Notch ligand genes whose deletion causes developmental changes in lymphopoiesis and myelopoiesis are shown in cells of the three hematopoietic compartments - bone marrow, thymus and spleen - based on data from single cell RNA-seq or microarray experiments reported in the ImmGen Skyline database (http://rstats.immgen.org/Skyline/skyline.html). NOTCH1 in ETP and DLL4 in thymic stroma, as well as NOTCH2 in MZ B and DLL1 in splenic stroma, are also shown as proteins.

*Notch2* is expressed in Pro B and Pre B cells of the bone marrow, and in the different B cell subsets of spleen (Garis and Garrett-Sinha, 2021). However, conditional deletion of *Notch2* using *Mx1*-Cre primarily inhibits development of MZ B cells and their precursors in spleen (Saito et al., 2003). DLL1 in splenic fibroblasts is the Notch ligand responsible for stimulating Notch signaling *via* NOTCH2 to generate MZ B cells in spleen (Sheng et al., 2008). Plasticity in this differentiation has recently been revealed in the generation of MZ B cells from follicular B (Fo B) cells (Lechner et al., 2021). A diagram of bone marrow HSC differentiation into lymphoid and myeloid cells in thymus and spleen including expression of Notch receptor, Notch ligand and Notch-related glycosyltransferase genes from the ImmGen Project (Immunological Genome Project, 2020) is shown in Figure 3.

### Notch target genes in lymphopoiesis and myelopoiesis

Determining genes altered in expression following deletion of Notch pathway members in HSCs or progenitors must be performed in the earliest lineage in which the gene is expressed because an essential requirement for Notch signaling may mean that certain cell populations are not generated or are contaminated by cells that differentiate along aberrant pathways. For example, deletion of *Notch1* using *Mx1*-Cre does not appear to perturb bone marrow cell populations derived from HSCs, but blocks T cell development at an early stage, and causes T cell progenitors to differentiate into B cells and myeloid cells in the thymus (Radtke et al., 1999; Feyerabend et al., 2009). Consequently, gene expression analysis of CD4/CD8 double-negative (DN) thymocytes lacking *Notch1* which have become B cells and myeloid cells, would not provide insights into genes related to T cell development. Interestingly, deletion of RBP-Jκ in HSCs using *Vav1*-Cre or *Mx1*-Cre does not affect Notch target gene expression in HSCs, and actually increases expression of classic Notch target genes like *Hes1* and *Hes5* in megakaryocyte, erythroid and myeloid bone marrow progenitors (Duarte et al., 2018). However, investigations of thymus-seeding progenitors in bone marrow by an elegant gene rescue approach revealed requirements for RBP-Jκ and Notch signaling in the generation of the MPP4/LMPP lymphoid progenitor population in bone marrow (Chen et al., 2019). Thus, Notch signaling is required for generation of thymus-seeding progenitors in bone marrow. In the case of Notch target *Hes1*, conditional knockout with *Mx1*-Cre does not affect HSC or HSPC numbers but causes a major block in T cell development in thymus, as expected if Notch signaling in inhibited (Wendorff et al., 2010). However, MZ B cell numbers in spleen are not altered following the loss of *Hes1* in HSCs (Wendorff et al., 2010). Deletion of *Hes1* using *Vav1*-Cre also showed that hematopoiesis was not obviously altered. However, stress hematopoiesis was ineffective in stem cells lacking *Hes1,* which exhibit an exhausted phenotype (Ma et al., 2020).

### Strategies to identify roles for O-glycans in hematopoiesis

As mentioned above, Notch receptors and ligands are transmembrane glycoproteins with an ECD comprising numerous EGF repeats (Figures 2C,D). Each EGF repeat consists of ∼40 amino acids, including six conserved cysteines which form three disulfide bonds. EGF repeats may contain consensus sequences for the addition of fucose (Fuc), glucose (Glc) or N-acetylglucosamine (GlcNAc) to Ser or Thr to initiate an O-Fuc, O-Glc or O-GlcNAc glycan (Haltiwanger et al., 2022). Glycosyltransferases that transfer an initiating sugar reside in the ER - POFUT1 transfers Fuc from GDP-Fuc, POGLUT1/2/3 transfer Glc from UDP-Glc, and EOGT transfers GlcNAc from UDP-GlcNAc. O-fucose on an EGF repeat may be extended by Fringe GlcNAc-transferases LFNG, MFNG or RFNG, and the GlcNAc may be further extended by Gal and sialic acid (Sia). O-glucose may be extended by xylose (Urata et al., 2020) and O-GlcNAc may be extended by Gal and Sia (Ogawa et al., 2020). To date, experiments aimed at defining roles for O-glycans on EGF repeats in immune cell development have been performed by manipulating the O-fucose glycans by deleting or overexpressing *Pofut1*, *Lfng*, *Mfng* or *Rfng* (Figure 2A). Only approximately 100 proteins contain EGF repeats that may be modified by these O-glycans (Rampal et al., 2007). The four Notch receptors and five Notch ligands comprise almost 10% of these substrates. However, ascribing phenotypic changes observed after deletion or overexpression of one of these glycosyltransferases to changes in Notch signaling depends on whether the phenotype mimics that of mutants deleted for, or overexpressing, other Notch pathway members; or on the mutation causing defective activation of Notch receptors by ligand-induced cleavage to generate NICD; or on the altered expression of known Notch signaling target genes. To directly determine if loss of one or more O-glycans on a particular Notch receptor or Notch ligand provides the basis of a glycosylation-defective phenotype, it is necessary to mutate individual Ser/Thr residues to preclude glycosylation in a specific EGF repeat. This, however, comes with caveats, since the alternative amino acid chosen can affect the outcome, as described below.

### O-Fucose glycans and Notch signaling in lymphopoiesis and myelopoiesis

Deletion of *Pofut1* in mice results in embryonic lethality with a phenotype typical of defective Notch signaling (Shi and Stanley, 2003; Okamura and Saga, 2008). Conditional deletion of *Pofut1* in bone marrow HSCs using *Mx1*-Cre or *Vav1*-Cre causes changes in lymphoid and myeloid cell differentiation (Yao et al., 2011; Yu et al., 2015). HSC progenitors (Lineage minus (Lin-)Sca1+cKit + bone marrow cells termed LSK) lacking *Pofut1* exhibit a 3-6-fold reduction in the binding of soluble Notch ligands DLL1-Fc and DLL4-Fc, a major decrease in ligand recognition, and a slight reduction of ∼30% in cell surface expression of NOTCH1 and NOTCH2 (Yao et al., 2011), indicating a minor effect on Notch receptor trafficking. Myeloid cells in bone marrow were increased, whereas T cell production in thymus, and MZ B cells in spleen were decreased, as expected if the loss of O-fucose glycans causes defective Notch signaling *via* both NOTCH1 and NOTCH2. In addition, expression of Notch target genes *Dtx1* and *Hes1* was depressed in co-cultures of LSK cells with OP9 stromal cells under conditions that induce differentiation to T cells. Bone marrow transplantation revealed that the reduced lymphopoiesis and altered myelopoiesis following conditional deletion of *Pofut1* arose largely from cell-autonomous effects in HSCs and HSC progenitors (HSPC). The overall findings validated conclusions of earlier experiments that characterized defective myelopoiesis in mice with a global knockout of *Gmds*, an enzyme required for the synthesis of GDP-Fuc, the substrate of POFUT1 (Zhou et al., 2008). Loss of O-fucose on Notch receptors, proposed as the basis of changes in myelopoiesis in the *Gmds* [−/−] mice, is consistent with the Notch-defective phenotype of mice lacking *Pofut1* the enzyme that transfers O-fucose to all Notch receptors and ligands (Yan et al., 2010). Interestingly, conditional deletion of the downstream transcription factor RBP-Jκ that transduces Notch signaling *via* all combinations of Notch receptors and ligands, gives more severe reductions in lymphoid and myeloid cells than deletion of *Pofut1* in HSC (Yu et al., 2015), suggesting that other O-glycans on Notch receptors might support Notch signaling in the absence of O-fucose glycans. We recently addressed this question by generating mice lacking *Eogt* and O-GlcNAc glycans as well as being conditionally deleted for *Pofut1* in HSCs, as described below (Tanwar and Stanley, 2022).

An alternative approach to deleting *Pofut1* is to pinpoint functions of O-fucose in NOTCH1 by mutating key O-fucose sites in EGF repeats of the ECD. The first example in mouse was mutation of the O-fucose site in EGF12 of NOTCH1 from Thr to Ala (Ge and Stanley, 2008). EGF12 is within the Notch ligand binding domain, and we now know that the O-fucose in EGF12 of NOTCH1 is recognized directly by Notch ligands (Luca et al., 2015; Luca et al., 2017). Homozygotes with the EGF12 mutation termed *Notch1[12f/12f],* are viable and fertile in a mixed genetic background. However, they exhibit effects typical of reduced NOTCH1 signaling in T cell development. Thymus size and thymocyte numbers were reduced ∼50%, and T cell progenitors, CD4 or CD8 single-positive (SP) and CD4/CD8 double-positive (DP) T cells were reduced in thymus. In addition, *Notch1[12f/12f]* thymocytes exhibit reduced expression of activated NOTCH1 (NICD), and reduced levels of Notch targets *Hes1* and *Dtx1*. Binding of Notch ligand DLL1 to thymocytes is also reduced. The defective T cell phenotype is cell autonomous following transplantation of *Notch1[12f/12f]* bone marrow. Therefore, loss of one critical fucose in EGF12 of NOTCH1 significantly weakens signaling *via* NOTCH1, even though O-fucose is probably present on 19 other EGF repeats with an O-fucose consensus site (Figure 2C). This hypomorphic phenotype is surprisingly maintained in mice overexpressing *Lfng* in T cells (Visan et al., 2010).

The question of whether loss of O-Fuc from EGF12 in NOTCH1 due to conversion of Thr to Ala or the amino acid mutation itself causes the T cell phenotype was not addressed in the mouse mutant. However, it was previously investigated in cell-based Notch signaling assays. In these experiments, the Thr in NOTCH1 EGF12 was changed to Ala to abrogate O-Fuc addition or, alternatively, changed to Ser which accepted O-fucose (Shi et al., 2007). The Thr to Ala mutation in EGF12 inactivated NOTCH1 signaling in the cell-based assay, but the Thr to Ser mutation did not, consistent with O-fucose being required in EGF12 for NOTCH1 signaling. The inhibition of NOTCH1 signaling in the Thr to Ala mutant construct was essentially complete in these experiments. Thus, it was surprising that *Notch1[12f/12f]* homozygous mice were viable (Ge and Stanley, 2008). The explanation turned out to be genetic background. When the *Notch1[12f]* mutation was backcrossed into the C57BL/6J background for >10 generations, *Notch1[12f/12f]* homozygotes died at E11.5 (Varshney et al., 2019). It seems that a modifying activity in the initial mixed genetic background rescued *Notch1[12f/12f]* from embryonic lethality.

These findings highlight the complexities of interpreting the results of point mutations that preclude a glycosylation reaction. In another example, a mutation converting an Asn which receives an N-glycan in the enzyme POFUT1 to Leu was found to reduce POFUT1 activity. However, substitution of the Asn with Gln that also could not receive an N-glycan, restored POFUT1 activity (Takeuchi et al., 2018b). Therefore, certain amino acids at that position are required for POFUT1 activity, but the N-glycan attached to Asn is not required for POFUT1 to be active. In another example, mutation of Thr to Ala in the EGF repeat of Cripto inhibits Cripto signaling. However, mutation to Ser, which could receive an O-fucose, also inhibits Cripto signaling, leading to the conclusion that the Thr, and not the O-fucose, is necessary for Cripto signaling (Shi et al., 2007). The take-home message from these experiments is that all caveats should be explored to enable interpretation of mutant phenotypes in relation to glycosylation status.

### Fringe GlcNAc-transferases in lymphopoiesis and myelopoiesis

The first investigation of roles for Fringe GlcNAc-transferases in T cell development showed that overexpression of *Lfng* under the control of the *lck* promoter that is initially expressed in T cell progenitors, blocks T cell development and leads to a large population of B cells in the thymus (Koch et al., 2001). This result is consistent with numerous subsequent experiments showing that B cells and myeloid cells are generated from thymic T cell precursors blocked in Notch signaling by a variety of strategies (Brandstadter and Maillard, 2019). Most interesting is the basis of the block caused by overexpressing *Lfng* in thymocytes, which might be expected to enhance rather than block Notch signaling because LFNG modification of Notch receptors generally enhances signaling induced by DLL ligands (Hicks et al., 2000). Elegant experiments showed that mature thymic T cells overexpressing *Lfng* compete with early T cell progenitors for binding to DLL4-expressing thymic stroma, and thereby prevent access of T cell progenitors to Notch ligands that stimulate Notch signaling and promote differentiation along the T cell lineage (Visan et al., 2006). Overexpression or loss of *Lfng* in fetal liver-derived HSC confirmed that *Lfng* is required for optimal T cell development (Tsukumo et al., 2006). Loss of *Lfng* from CD4/CD8 double-negative 3 (DN3) T cell progenitors inhibits their proliferation and causes them to differentiate prematurely to CD4/CD8 double-positive (DP) T cells (Yuan et al., 2011). Thus, extension of O-fucose on NOTCH1 in pre-T cells by LFNG enhances Notch ligand binding to Notch receptors and Notch signaling, thereby prolonging proliferation of the pool of DN3 T cell progenitors and promoting the production of mature thymic T cells. The role of LFNG is therefore to enhance the strength of Notch signaling in T cell progenitors, a necessary boost required for optimal production of T cells in thymus (Yuan et al., 2011). However, overexpression of *Lfng* under the *lck* promoter could not fully rescue reduced T cell production in the *Notch1[12f/12f]* thymus (Visan et al., 2010). This result indicates that extension of the O-fucose at EGF12 of NOTCH1, regardless of potentially enhanced extension of O-fucose at other EGF repeats due to prolonged overexpression of *Lfng*, is required for optimal Notch signaling in T cell progenitors. In these experiments, *Lfng* expression was manipulated only in Notch receptor-expressing T cells. Requirements of *Lfng* for Notch ligand functions in thymic stroma were not addressed. However, there are cellular contexts in which effects of Fringe modification of canonical Notch ligands have been identified in other systems (for example (Bochter et al., 2022) and (Kadur Lakshminarasimha Murthy et al., 2018)), and it will be important to examine this issue and the role of each FNG during hematopoiesis in future.

Roles for each of the Fringe GlcNAc-transferases in T and B cell development were revealed in mice inactivated for all three *Fng* genes compared to mice expressing a single *Fng* gene from one allele (Song et al., 2016). Loss of all three Fringe GlcNAc-transferases leaves Notch receptors and Notch ligands with EGF repeats carrying O-fucose that cannot be extended (Figure 2A). Triple knockout (*Fng* tKO) mice have reduced DLL4-Fc binding to DN T cell progenitors, and reduced frequencies of early thymic progenitors (ETP), DN2 T cell progenitors, and double-positive (DP) T cells (Song et al., 2016). Increased frequencies of CD4 and CD8 single-positive (SP) T cells in the thymus were a novel characteristic of *Fng* tKO thymus that could indicate delayed exit of mature SP T cells from the thymus. The *Fng* tKO phenotype is transferred cell-autonomously by bone marrow. Substantial rescue of T cell development is achieved in mice expressing only *Lfng*, only *Mfng,* or only *Rfng* from a single allele. Thus, each *Fng* contributes to Notch signaling in thymic T cells and promotes optimal T cell development.

In spleen, both LFNG and MFNG are required for MZ B cell development (Tan et al., 2009). Deficiency of either one reduces the number of MZ B cells in spleen. In mice expressing no *Fng* genes, there is a reduced frequency of MZ B cells as expected, and a corresponding increase in Fo B and MZ P precursor B cells. CD4^+^ and CD8^+^ splenic T cells are also decreased. By contrast, granulocyte numbers are increased (Song et al., 2016). All changes are consistent with reduced Notch signaling and show that Fringe modification is necessary for optimal T and B cell and granulocyte production. In terms of individual *Fng* genes, *Lfng* was able to rescue the frequency of T and MZ B cells in the absence of both *Mfng* and *Rfng*. *Mfng* or *Rfng* expressed from a single allele accomplished a less complete rescue (Song et al., 2016). There are also functional consequences for T cell responses in mice lacking all Fringe activities. *Fng* tKO splenocytes stimulated by anti-CD3/CD28 beads or lipopolysaccharide (LPS) or Concanavalin A exhibit reduced proliferation (Song et al., 2016). In addition, naïve T cells and effector memory T cells were reduced in *Fng* tKO spleen.

### O-GlcNAc glycans in hematopoiesis

Mice lacking *Eogt* and O-GlcNAc are viable and fertile but exhibit mildly defective Notch signaling during retinal angiogenesis (Sawaguchi et al., 2017). This laboratory is investigating effects of deleting *Eogt* and O-GlcNAc glycans on hematopoiesis, lymphopoiesis and myelopoiesis in *Eogt* null mice. In *Eogt*[−/−] thymus, T cell progenitors and DP T cell numbers were reduced, while SP T cells were slightly increased (Tanwar and Stanley, 2022), as observed when all three *Fng* genes are deleted (Song et al., 2016). Consistent with Notch signaling being inhibited due to the loss of *Eogt* and O-GlcNAc glycans, B cells and myeloid cells were increased in thymus, and Notch target genes *Hes1* and *Il2ra* were reduced in expression. In spleen, CD4^+^ and CD8^+^ T cells were not reduced, but NK T cells were reduced along with myeloid cells. Surprisingly, B cell subsets were slightly increased. The *Eogt* null hematopoietic phenotype was largely cell-autonomous following bone marrow transfer.

The fact that loss of *Eogt* caused effects on T and B cell development consistent with reduced Notch signaling suggested that O-GlcNAc glycans might support Notch signaling in the absence of *Pofut1* and O-fucose glycans. This hypothesis was tested by deleting *Pofut1 via Vav1*-Cre in HSCs, in the presence and absence of *Eogt*. Deletion of *Pofut1* in HSCs caused myeloid hyperplasia and a drastic reduction in T cell development and MZ B cell production, as described previously (Yao et al., 2011; Yu et al., 2015). The additional deletion of *Eogt* enhanced the Notch-defective phenotype in bone marrow, thymus and spleen (Tanwar and Stanley, 2022). Thus, EOGT and O-GlcNAc glycans are required for optimal Notch signaling in the development of myeloid and lymphoid cells. In addition, O-GlcNAc glycans promote Notch signaling in the absence of O-fucose glycans and partially rescue HSCs and descendants that lack *Pofut1*.

### Congenital diseases affected in EGF repeat modification by O-glycans

Congenital disorders of glycosylation (CDG) are rare in human populations, but there are many distinct diseases. At latest count, congenital mutations have been reported in >140 different genes involved in glycosylation (Sosicka et al., 2021; Lefeber et al., 2022). Of the glycosyltransferases that modify Notch EGF repeats and promote hematopoiesis, deleterious mutations compatible with life in humans have been identified in *POFUT1, LFNG*, and *EOGT*. Mutations in *POFUT1* cause an autosomal dominant disease termed Dowling Degos Disease 2 (DDD2) which causes skin hyperpigmentation and follicular skin lesions (Li et al., 2013). However, genome sequencing and mosaic analysis have identified mutations in *POFUT1* that are not classified as a classical DDD2 phenotype. A recessive mutation in a person with microencephaly and developmental delay was found to correlate with weak POFUT1 activity (Takeuchi et al., 2018b). Another recessive *POFUT1* mutation expands the DDD2 phenotype with new features such as dermatitis (Atzmony et al., 2019). Given the requirement for POFUT1 for lymphopoiesis and myelopoiesis described above, patients with weak POFUT1 may have deficiencies in innate and adaptive immunity. Skin lesions observed in these patients may reflect reduced activity of the immune system in skin (Nguyen and Soulika, 2019).

Humans with inactivating mutations in *LFNG* have Spondylocostal Dystosis Type 3 (SCDO3), an autosomal recessive disease that gives rise to malformation of the skeleton during embryogenesis (Sparrow et al., 2006; Dunwoodie, 2009). Deletion of *Lfng* in the mouse causes severe skeletal damage with loss of the tail (Evrard et al., 1998; Zhang and Gridley, 1998), and may be embryonic lethal, depending on genetic background (unpublished observations). LFNG promotes T and MZ B cell development, but is partially compensated for by MFNG and RFNG (Tan et al., 2009; Song et al., 2016). Consequently, effects of the loss of LFNG activity on immune cell development would be expected to be relatively minor in humans, and thus impacts on the immune response of SCDO3 patients would also presumably be minor.

Autosomal recessive *EOGT* mutations in humans give rise to a disease termed Adams-Oliver Syndrome type 4 (AOS4), which is characterized by aplasia cutis congenita of the scalp, limb and digit malformations, and in some cases, vascular and cardiac defects (Schröder et al., 2019). The O-GlcNAc-transferase activity of EOGT containing AOS4 mutations is greatly reduced (Ogawa et al., 2015). Considering that AOS4 disease varies in severity and that AOS4 patients may live to adulthood, and given the defective T and altered B cell development observed in *Eogt* null mice (Tanwar and Stanley, 2022), *EOGT* mutations would be expected to affect immune cell development in humans with AOS4, and potentially to compromise certain immune responses. Further experiments on innate and adaptive immune responses in *Eogt* null mice will assist in predicting immune response concerns in AOS4 patients.

### Lymphoid and myeloid malignancies with altered Notch signaling

Notch signaling was first discovered in mammals in acute T lymphoblastoid leukemia (T-ALL) which arises in many cases from a chromosomal translocation that causes constitutive activation of *NOTCH1* (Ellisen et al., 1991). Subsequently, activating mutations in the *NOTCH1* gene were found to cause >50% of T-ALLs (Weng et al., 2004). A proportion of these mutations occur in the NOTCH1 heterodimerization (HD) domain just above the plasma membrane (Figure 1) and render NOTCH1 active independently of Notch ligand stimulation. However, Notch ligands may theoretically stimulate such activated NOTCH1 mutants, since the ECD with its attached O-glycans is present. Another class of *NOTCH1* mutations in T-ALL occurs in the PEST domain and functions by prolonging the life of NICD in the nucleus (Weng et al., 2004). In this case, Notch ligand induces Notch signaling which is sustained due to the lack, or inactivation of, the PEST domain on NICD. Several other types of leukemia also arise from PEST domain mutations in *NOTCH1*, including chronic lymphocytic leukemia (CLL), and Diffuse Large B cell lymphoma (Siebel and Lendahl, 2017). While there have been no reports of an involvement of O-glycan synthesis in leukemogenesis or other blood disorders with disrupted Notch signaling, amplification of the *POFUT1* gene has been observed in certain cases of myeloid malignancy (Mackinnon et al., 2010). Thus, loss of the long arm of one copy of chromosome 20, that occurs in cases of acute myeloid leukemia (AML) and myelodysplastic syndrome (MDS) and is presumed to assist leukemogenesis by removal of a tumor suppressor-like gene, is often accompanied by amplification of the remaining portion of chromosome 20 (20q11.2). The *POFUT1* gene resides in the smallest amplified domain at 20q11.21, along with the *HCK, TM9SF4* and *PLAGL2* genes. Investigation of a functional role for *POFUT1* gene amplification in models of AML and MDS might reveal enhanced Notch signaling due to increased O-fucosylation of Notch receptors, or ligands. A similar amplification of chromosome region 20q11.21 is seen in colon cancer (Chabanais et al., 2018). However, the website Tumor Portal sponsored by the BROAD Institute (http://www.tumorportal.org/), which reports expression levels and mutations across many cancer types, notes that neither *POFUT1*, *LFNG* nor *EOGT* are “near significance” for mutation or expression changes in any lymphoid or myeloid tumor type.

## Discussion

The development of HSCs, the many HSPCs, myeloid, and lymphoid cells reflects a complicated set of lineage decisions requiring co-ordination at the molecular and cellular levels. Notch signaling is a key regulator of several stages of differentiation through different combinations of Notch receptors, Notch ligands and glycosyltransferases that modify extracellular domain EGF repeats (Figure 1). The focus of this review is on Notch receptors, ligands and glycosyltransferase genes that have been manipulated to uncover specific roles in hematopoiesis, myelopoiesis and lymphopoiesis for regulation by glycosylation. Table 1 summarizes key experimental systems and findings discussed in the text. NOTCH1 is essential for T cell development and DLL4 is required, but *Dll4* deletion in thymic stroma can be rescued by *Dll1*. NOTCH2 is essential for MZ B cell development and DLL1 is also required. It is not known whether DLL4 could substitute for DLL1 in splenic stroma. JAG1 and JAG2 have roles in the generation of HSCs from hemogenic endothelium and the glycosyltransferases have various roles. Deletion of *Poglut1* is embryonic lethal (Fernandez-Valdivia et al., 2011), and to date there have been no reports on the consequences of conditionally deleting *Poglut1* and O-glucose glycans in HSCs, or other immune cells, or stromal cells. However, the effects of knocking out *Pofut1*, *Lfng*, *Mfng*, *Rfng* and *Eogt* on myeloid and lymphoid cell development have been investigated. Deletion of *Pofut1* in HSCs causes altered generation of B cells, myeloid cells, and thymus-seeding progenitors in bone marrow. *Pofut1* null T cells that enter the thymus are mainly converted to B cells and myeloid cells, and T cell development is blocked. A similarly dramatic phenotype is observed when *Lfng* is overexpressed in thymic T cells. Loss of *Lfng* causes a relatively mild reduction in T cell development which is ameliorated by the presence of *Mfng* and/or *Rfng.* In spleen, *Lfng* and *Mfng* are required together for optimal MZ B-cell development. Mice lacking *Eogt* exhibit altered cell numbers in bone marrow B cells and myeloid cells, thymic T cells and MZ B cells in spleen. As structural methods develop it should become possible to analyze the actual changes in O-glycans occupancy and extension among Notch receptors in mouse models. For now, the combined data suggest that sugars added to the EGF repeats of Notch receptors and ligands play a part in optimizing the generation of appropriate numbers of cells during hematopoiesis, myelopoiesis and lymphopoiesis. Reduced ligand binding to progenitor cells identifies one mechanism for reduced Notch signaling strength. Consistent with this, reduced ligand binding is observed in DN T cells expressing NOTCH1[12f] in which there is no O-fucose attached to EGF12, or no expression of LFNG, MFNG or RFNG. LSK cells in bone marrow show reduced Notch ligand binding when POFUT1 is absent. However, loss of EOGT does not significantly affect soluble Notch ligand binding to DN T cells, although Notch signaling is reduced, indicating that binding strength may be impaired. Since glycosyltransferases that modify Notch promote immune cell development, people with deleterious mutations in one of these glycosyltransferase genes may have immune response defects. Functional consequences were observed in responses of activated T cells lacking Fringe activity, but many more studies are needed to uncover potential problems with immune responses. In immune cell cancers, inhibition of Notch signaling might be achieved by inhibition of glycosylation. One example of this approach used incorporation of a chemically modified fucose into EGF repeats to block Notch signaling (Schneider et al., 2018). A better understanding of how Notch signaling is regulated and becomes dysregulated will enable promotion or inhibition of signaling in order to optimize immune responses in humans. Other signaling pathways such as those directed by cytokines or NFκB also influence lymphoid and myeloid cell development (Tsaouli et al., 2020). However, these pathways should not be directly affected by inactivation of any of the EGF repeat-modifying glycosyltransferases discussed in this review, unless a critical member of the signaling pathway contains one or more EGF repeats with O-glycans required for the function of the parent molecule.

**TABLE 1:** Notch Signaling-Defective Mutants and Hematopoiesis.

Gene	Mouse Models	Bone marrow	Thymus	Spleen	References
*Notch1*	*Notch1* ^*lox/lox*^ *Mx1-*Cre	BM transfer gave T cell defects, spleen and lymph nodes populated with *Notch1* ^-/-^ cells	T cells greatly reduced. Increased B cells and myeloid cells	No effects on B cells, erythroid or myeloid cells	Radtke et al. (1999), Wilson et al. (2001)
	*Notch1[12f/12f]*	BM transfer of T cell phenotype	∼50% less thymocytes, reduced T cells. No increase in B-cells.	Not investigated	Ge and Stanley, (2008)
*Notch2*	*Notch2* ^*-/-*^ *Mx1*-Cre	Normal HSC function	No effects on T cell development	Largely abolished MZ B cells and precursors	Kumano et al. (2003)
*Notch1, Notch2*	*Notch1* ^*-/-*^ *Notch2* ^*-/-*^ HSC deletion by CRISPR/Cas9	Not applicable	T cell development on OP9 cells reduced in N1^-/-^N2^-/-^ compared to N1^-/-^ HSC	Not applicable	Romero-Wolf et al. (2020)
*RBPJ*	*RBPJ-k* ^*f/f*^ *Mx1*-Cre	Dysregulated HSPC differentiation	Reduced T cell development Increased B cells and myeloid cells	No change in B cells	Han et al. (2002), Yu et al. (2015)
	*RBPJ-k* ^*f/f*^ *Vav1*-Cre	Reduced LMPP/MPP4 progenitors Increased GMP	Reduced T cell development Increased B cells and myeloid cells	Dysregulated B cell development Myeloid hyperplasia	Chen et al. (2019), Lakhan and Rathinam, (2021)
*Dll1*	*Dll1* ^*lox/lox*^ *Mx1*-Cre	No change in hematopoiesis	No change in T cell development	MZ B cells largely absent	Hozumi et al. (2004)
*Dll4*	*Dll4* ^*f/f*^ *Foxn1*-Cre with *Dll1or Dll4 transgene in ROSA-26 locus*	Not applicable	Loss *Dll4* reduces T cell development. *Dll1* or *Dll4* transgene rescued	Not applicable	Hozumi et al. (2008), Hirano et al. (2022)
*Jag1*	*Jag1* ^*lox/lox*^ *Mx1*-Cre	No effect on HSC or HSPC	No phenotype	No phenotype	Mancini et al. (2005)
	*Jag1* ^*(ECKO)*^ VE-cadherin-Cre	Reduced LT-HSC, LSK cells; increased stress sensitivity (LSK)	Not investigated	Not investigated	Poulos et al. (2013)
	*Jag1* ^*-/-*^ embryos	Block in HSC generation from AGM	Not investigated	Not investigated	Gama-Norton et al. (2015)
*Jag2*	*Jag2* ^*(ECKO)*^ VE-cadherin-Cre	Reduced HSPC and T cells following myeloablation	No effects	No effects	Guo et al. (2017)
*Lfng*	PrL*Lfng* transgenic (Proximal *Lck* promoter)	Not investigated	T cell development blocked at pro-T stage. Increased pro- B and more mature B cells	No apparent effects	Koch et al. (2001), Visan et al. (2006)
*Lfng, Notch1*	PrL*Lfng:Notch1[12f/12f]*	Not investigated	Ameliorated PrL*Lfng* phenotype	Not investigated	Visan et al. (2010)
*Lfng, Mfng*	*Lfng* ^*-/-*^; *Mfng* ^*-/-*^ *Lfng* ^*-/-*^; *Mfng* ^*-/-*^ dKO	BM transfer of spleen phenotype from all mutants	Not investigated	Reduced MZ B cells in single and double KO. *Lfng* and *Mfng* complementary	Tan et al. (2009)
*Lfng,Mfng, Rfng*	*Fng* tKO	BM transfer of thymus and spleen phenotypes	Retarded T-cell development No increase in B cells Corrected by each *Fng* alone	Reduced MZ B and Fo B cells. Increased MZP, neutrophils and NK T cells. Reduced activation of T cells	Song et al. (2016)
*Pofut1*	*Pofut1* ^*F/F*^ *Mx1-*Cre *Pofut1* ^*F/F*^ *Vav1*-Cre	Myeloid hyperplasia, reduced B cells. Increased GMP, reduced CMP, MEP. BM transfer of thymus and spleen phenotypes	Blocked T cell development Increased B cells and myeloid cells	Reduced T cells, MZ B cells Increased myeloid cells	Yao et al. (2011), Yu et al. (2015), Tanwar and Stanley, (2022)
*Eogt*	*Eogt* ^*-/-*^	Increased B cells and myeloid cells. Phenotype partially transferred by BM	Reduced T cell development Increased B cells and myeloid cells	Increased B cells Reduced NK T cells and myeloid cells	Tanwar and Stanley, (2022)
*Eogt, Pofut1*	*Eogt^-/-^Pofut1^F/F^Vav1-Cre*	Increased ST-HSC, LSK, HSPC, GMP Reduced MPP, CLP, CMP, MEP, T cells, B cells	T cell development abolished Increased B cells, myeloid cells, NK cells	Reduced Fo B, MZ B, mature B cells and T cells. Increased MZ P and myeloid cells	Tanwar and Stanley, (2022)
